# Recombinant full-length *Bacillus Anthracis* protective antigen and its 63 kDa form elicits protective response in formulation with addavax

**DOI:** 10.3389/fimmu.2022.1075662

**Published:** 2023-01-12

**Authors:** Shikhar Sharma, Vanndita Bahl, Gaurav Srivastava, Risha Shamim, Rakesh Bhatnagar, Deepak Gaur

**Affiliations:** ^1^ Laboratory of Malaria & Vaccine Research, School of Biotechnology, Jawaharlal Nehru University, New Delhi, India; ^2^ Department of Oncology Science, University of Oklahoma Health Science Center, Oklahoma City, OK, United States; ^3^ Food Technology Division, Bhabha Atomic Research Centre, Mumbai, Maharashtra, India; ^4^ School of Life Sciences, Jawaharlal Nehru University, New Delhi, India; ^5^ Molecular Biology and Genetic Engineering Laboratory, School of Biotechnology, Jawaharlal Nehru University, New Delhi, India

**Keywords:** protective antigen, vaccine, adjuvants, *Bacillus anthracis*, addaVax

## Abstract

**Introduction:**

*Bacillus anthracis* is the causative agent for the lethal disease anthrax, primarily affecting animals and humans in close contact with an infected host. The pathogenicity of *B. anthracis* is attributed to the secreted exotoxins and their outer capsule. The host cell-binding exotoxin component “protective antigen” (PA) is reported to be a potent vaccine candidate. The aim of our study is to produce several PA constructs and analyze their vaccine potential.

**Methods:**

We have designed the various subunit, PA-based recombinant proteins, i.e., full-length Protective antigen (PA-FL), C-terminal 63 kDa fragment (PA63), Protective antigen domain 1-domain 4 chimeras (PA-D1-4) and protective antigen domain 4 (PA-D4) and analyzed their vaccine potential with different human-compatible adjuvants in the mouse model. We have optimized the process and successfully expressed our recombinant antigens as soluble proteins, except full-length PA. All the recombinant antigen formulations with three different adjuvants i.e., Addavax, Alhydrogel, and Montanide ISA 720, were immunized in different mouse groups. The vaccine efficacy of the formulations was analyzed by mouse serum antigen-specific antibody titer, toxin neutralization assay, and survival analysis of mouse groups challenged with a lethal dose of *B. anthracis* virulent spores.

**Results:**

We have demonstrated that the PA-FL addavax and PA63 addavax formulations were most effective in protecting spore-challenged mice and serum from the mice immunized with PAFL addavax, PA-FL alhydrogel, PA63 addavax, and PA63 alhydrogel formulations were equivalently efficient in neutralizing the anthrax lethal toxin. The higher levels of serum Th1, Th2, and Th17 cytokines in PA-FL addavax immunized mice correspond to the enhanced protection provided by the formulation in challenged mice.

**Discussion:**

We have demonstrated that the PA-FL addavax and PA63 addavax formulations exhibit equivalent efficiency as vaccine formulation both in a mouse model of anthrax and mammalian cell lines. However, PA63 is a smaller antigen than PA-FL and more importantly, PA63 is expressed as a soluble protein in *E.* coli, which imparts a translational advantage to PA63-based formulation. Thus, the outcome of our study has significant implications for the development of protective antigen-based vaccine formulations for human use against the lethal disease anthrax.

## Background

Anthrax is one of the most lethal zoonotic diseases caused by the sporulating bacterium *Bacillus anthracis.* It spreads mainly *via* spores that are tolerant to hostile environments and survive for years (1, 2). Under nutrient-rich conditions such as the host body, they germinate into the vegetative bacilli and divide rapidly. Its rapid division is complemented by the potential to evade immune response due to its two primary virulence factors i) a weakly immunogenic capsule that confers resistance towards phagocytosis, and ii) anthrax toxins, which disrupt various host physiological functions and flattens the immune response (3). The *Bacillus anthracis* infection leads to extensive bacteremia and toxemia (1). Its natural infections in humans are rare; however, its intentional spread is a potential bioterrorism agent and can cause severe health problems (4, 5).

*The B. anthracis* capsule is crucial for escaping the host’s innate immune response and hence provides the survival advantage to the encapsulated bacterium. *B. anthracis* capsule is composed of poly-γ-D-glutamate (PGA), which is negatively charged at physiological pH and eventually limits the binding of complement proteins, immunoglobulins, and acute phase proteins on bacterial surfaces (6, 7). Capsule being the primary virulence factor of *B. anthracis* and considering its crucial role in the establishment of infection, capsule targeting is considered a potential anthrax intervention strategy. There are several studies demonstrating the efficacy of capsule-conjugated vaccine candidates (8, 9). Anthrax toxin is a binary A-B type toxin that comprises a cell binding component i.e., protective antigen (PA), and two enzymatic toxigenic components, edema factor (EF) and lethal factor (LF). PA, In combination with LF and EF, forms lethal toxin and edema toxin, respectively. The protective antigen binds to the mammalian cells and acts as the translocase for the entry of LF and EF (10–12). The lethal factor is a Zn^+2^-dependent metalloprotease that inhibits protein mitogen-activated protein kinase kinases (MAPKKs), including MEK1, MEK2, and MKK3 in the host cells (13). p38 MAP kinase inactivation downstream of MKK3 dampens the reactive oxygen species (ROS) and TNF-α production in macrophages, thus affecting their inflammatory role (13, 14). Edema factor, which is a calmodulin (CaM) dependent adenyl cyclase (AC), increases the level of cAMP above the normal physiological level resulting in activation of cAMP-dependent protein kinase (PKA), guanine nucleotide exchange factors (GEFs) and ion channels. Aberrant activation of ion channels leads to edema resulting in membrane rupture, thus compromising host defenses (15, 16).

Structurally, protective antigen consists of four functional domains. N-terminal Domain 1 (residues 1-258) contains a furin protease cleavage site (RKKR) at residues 164-167 that leads to the cleavage of an N-terminal 20 kDa (residues 1-167) fragment (PA20). The remaining 63 kDa protein (PA63) heptamerize through monomeric interactions with ANTXR receptors on the host cell surface and forms the pre-pore for LF/EF translocation (17). PA63 is involved in the binding of LF and/or EF, while PA20 induces apoptosis in human peripheral blood leukocytes (18). Domain 2 (residues 259-487) has a β-barrel core structure that spans the membrane, and its pore mediates the translocation of LF and EF. Domain 2 has 5-9 histidine residues; protonation of these residues induces a conformational change resulting in membrane insertion. Domain 3 (residues 488-595) has four stranded β sheets and is involved in protein interactions within the oligomer. Domain 4 (residues 596-735) has a β sandwich with an Immunoglobulin-like fold. The immunoglobulin-like fold has an open loop between 4β9 and 4β10, like the CDR3 loop of immunoglobulin. This loop is a good mediator of interaction between PA and cellular receptors. PA63 heptamer is water-soluble at alkaline or neutral pH and becomes lipid-soluble at acidic pH (19–21).

The protective antigen is the common cell adhesion component of both lethal toxin and edema toxins, thus indispensable for anthrax pathogenicity (22). Blocking the binding of protective antigen with the host cell receptors, CMG2 and TEM8, would impede the intracellular entry of the toxins in the host cell. Therefore, blocking the interaction of PA with host cell receptors is a tailor-made strategy for developing preventive and therapeutic interventions such as vaccines and targeted drugs (23–27). Currently, attenuated vaccines (a Russian vaccine based on the STI-1 strain and a Chinese vaccine based on the A16R strain) and vaccine formulations based on non-encapsulated *B. anthracis* strains, e.g., the US Anthrax Vaccine Adsorbed (AVA) vaccine, and the UK Anthrax Vaccine Precipitate (AVP) vaccine are the most common anthrax vaccines. Despite the demonstrated efficacy of these vaccines, it may be difficult to standardize these vaccines regarding their toxin concentration and purity. Secondly, there is a continuous requirement of booster doses for these vaccines (28, 29). Therefore, to overcome the limitations of current vaccines, it is essential to develop the next generation of novel intervention strategies. Vaccines based on recombinant PA have already been developed by multiple groups, and their efficacy has also been shown to be promising. However, despite continuous efforts, it has not yet reached clinics (30, 31).

Production of a stable form of recombinant full-length protective antigen has been challenging due to its highly labile nature (32, 33). Therefore, our present study aims to produce the different constructs of recombinant protective antigen and analyze their functionality and immunogenic potential with already approved human-compatible adjuvants. Our idea is to conserve the host cell binding domain of protective antigen i.e., domain 4 in all the constructs so that immunization with these antigens could produce receptor-PA interaction-blocking antibodies. Secondly, all the antigens in our portfolio except domain 4 have LF/EF binding sites, so it could generate PA-LF/EF interaction-blocking antibodies. We produced four constructs spanning the whole Protective antigen i.e., full-length protective antigen (PA-FL), C-terminal 63 kDa protein (PA63), fusion construct of domain 1 and domain 4 (PA-D1-4), Protective antigen Domain 4 (PA-D4) and analyzed their immune potential with human-compatible adjuvants Alhydrogel, Addavax, and Montanide ISA 720 (34).

## Materials and methods

### Cloning, protein expression, and purification

Protective antigen (PA) subunit constructs were designed and expressed in *E. coli* strains. The protein construct produced were i) Full-length Protective antigen (PA-FL), ii) PA63, iii) Fusion construct of PA domain 1 and domain 4 (PA-D1-4) iv) Protective antigen Domain 4 (PA-D4) (**Table S3**) (UniProt accession number; Q68GS1). All the proteins were designed with a C-terminal hexahistidine tag. Respective genes were PCR amplified from the protective antigen clone in the pQE30 vector as a template (35). Primers for all the protective antigen constructs were designed and purchased from Sigma-Aldrich (**Table S1**).

PCR products encoding the respective PA constructs were digested with NdeI and XhoI (New England BioLabs, Beverly, MA) and inserted downstream of the T7 promoter in the *E. coli* expression vector pET-24b (Novagen, San Diego, CA) to get the plasmid pET24b with PA constructs clones (**Figure S1**). To optimize the expression of recombinant proteins in soluble cell fraction, various strains of *E. coli* were transformed with cloned pET24b for each construct and were used to check the expression of recombinant proteins. *E. coli* clones were cultured in an LB culture medium (Himedia). Culture conditions are summarized in **Table S2**. *E. coli* cultures were harvested by centrifugation at 3,000 g. Cell pellets were suspended in lysis buffer containing 50 mM Tris (pH 8.0), 300 mM NaCl, and 5% Glycerol and lysed by sonication. Soluble proteins were purified from the cell lysate supernatant by metal affinity chromatography using Ni-nitrilotriacetic acid (Ni-NTA) resin under the increasing concentration of imidazole (0-500 mM) in Tris-based buffer. Metal affinity-purified proteins were further purified to homogeneity by anion-exchange chromatography using a Q-Sepharose column (GE Healthcare, Piscataway, NJ). Purified recombinant proteins were characterized by SDS-PAGE and size exclusion chromatography (SEC; Superdex 200; GE Healthcare). The PA-FL inclusion bodies were washed, collected by centrifugation at 15,000 × *g*, and solubilized in a buffer containing 50 mM Tris (pH 8.0), 6 M guanidium HCl, and 300 mM NaCl. PA-FL was purified from solubilized inclusion bodies by metal affinity chromatography using Ni-NTA resin. Ni-NTA purified PA-FL was refolded by rapid dilution (0.1 mg/ml) in Tris-based buffer (pH 8.2), Comprising 440 mM sucrose, 550 mM L-arginine, and 264 mM NaCl. Refolded protein was dialyzed against Tris-based buffer (pH 8.2) containing 30 mM NaCl and 100 mM sucrose and further purified to homogeneity by anion-exchange chromatography using a Q-Sepharose column (GE Healthcare, Piscataway, NJ). Characterization of the purified protein was done by SDS-PAGE and size exclusion chromatography (SEC; Superdex 200; GE Healthcare).

### Trypsin digestion assay

The recombinant proteins PA-FL and PA-D1-4 were functionally characterized for their sensitivity toward trypsin. The recombinant proteins were treated with trypsin to analyze the cleavage of the PA domain 1 (RKKR sequence) and expose the binding site for LF/EF. Briefly, 50µg of protein were incubated with 1µl of trypsin (Sigma-Aldrich) at room temperature for 30 min. Post incubation, the protein samples were mixed with SDS-PAGE loading dye and boiled at 95°C for 10 minutes. Trypsin cleavage in domain 1 and production of 20 kDa fragment was analyzed using SDS-PAGE (17).

### Binding assay for recombinant protein to RAW 264.7 cells

All the Protective antigen recombinant constructs produced in the study have a conserved host cell binding region, i.e., Domain 4. Therefore, for the functional characterization of our recombinant proteins, we analyzed the binding of all the proteins on the surface of mammalian cells. Briefly, 2 x 10^6^ RAW 264.7 cells were seeded in the six-well plate in DMEM (Himedia) supplemented with 10% fetal bovine serum (Gibco) and incubated at 37°C with 5% CO_2_ until confluent. The recombinant protein was diluted in DMEM (without FBS) at a concentration of 5µg/ml, and 1 ml of protein was added to each well after washing the cells with sterile PBS. Cells incubated with recombinant LF (kind gift from Prof. Rakesh Bhatnagar) (35), and cells treated with only secondary antibodies were used as negative controls. The recombinant proteins were individually incubated with RAW 264.7 cells for 1 hour at 37°C with 5% CO_2_. The cells were washed thrice with sterile PBS and incubated with mice anti-PA-FL polyclonal serum (dilution 1:500 for 1 hour at 37°C). The cells were washed thrice with sterile PBS and incubated with goat anti-mouse IgG antibodies (Alexa Fluor 488; Invitrogen) for 1 hour at 37°C. Cells were washed thrice with sterile PBS and then washed with chilled PBS to detach RAW 264.7 from the culture plate surface. Recombinant protein binding to the cell surface was measured by flow cytometry (BD FACS ARIA).

### Cytotoxicity assay and toxin neutralization assay

The recombinant proteins, PA-FL and PA63 were functionally characterized for their capability to translocate lethal factor into the cell and induce cytotoxicity. Toxin forming assay was performed with recombinant PA-FL and lethal factor (LF) (35). Briefly, RAW264.7 cells were seeded in 96 well plates in DMEM (Himedia) supplemented with 10% fetal bovine serum (Gibco) and incubated at 37°C with 5% CO_2_ until confluent. PA-FL and LF were mixed in DMEM without FBS. Culture medium was aspirated from the cells, and 100µl of recombinant protein mixture was added to each well. Cells were incubated for 6 hours at 37°C with 5% CO_2_. The medium was aspirated, and 50 µl of MTT (3-(4,5-Dimethylthiazol-2-yl)-2,5-Diphenyltetrazolium Bromide) solution was added from stock solution (2mg/ml) and incubated at 37°C for 30 min. To dissolve the formazan crystals, 100µl dimethyl sulphoxide (DMSO) (Merck) was added, and optical density was measured at 590nm. The minimum lethal concentration of both PA-FL and LF was titrated. LF concentration was fixed at 3.0 µg/ml, and PA-FL concentration was titrated from 0.25 µg/ml to 3.0 µg/ml. The minimum lethal concentration of PA-FL was 1 µg/ml. Similarly, the minimum lethal concentration of LF was estimated by keeping PA-FL concentration at 1 µg/ml and titrating LF concentration from 0.25 µg/ml to 3.0 µg/ml. The minimum lethal concentration of LF was estimated to be 1.5 µg/ml. The estimated minimum lethal doses of both PA (1 µg/ml) and LF (1.5 µg/ml) (**Figure S3**) were used for setting up toxin neutralization by pooled mice sera. To assess the toxin-neutralizing efficacy of the sera from the immunized mice, a toxin-neutralization assay was performed. The day 42, pooled sera from the immunized mice group were serially diluted in DMEM (containing 10% FBS) from a dilution range of 1:100 to 1: 40,000. The mixture of recombinant PA and LF was incubated with different serum dilutions in DMEM for 1 hr. at 37°C. Protein-serum mixtures were added to the RAW264.7 cells and incubated for 6 hrs. at 37°C. The viability of the cells was assessed by MTT assay, as described earlier.

### Mice immunization and antibody quantification

BALB/c mice were obtained from National Centre for Laboratory Animal Sciences, NIN, Hyderabad, India, and were maintained in the animal holding room of the BSL3 laboratory. All the animal experiments, including spore challenge studies, were performed in compliance with the Institutional Animal Ethics Committee (Jawaharlal Nehru University) and Council for the Purpose of Control and Supervision of Experiments on Animals (CPCSEA, Ministry of Social Justice and Empowerment, Government of India). Groups of eight mice were immunized with antigen-adjuvant formulations. Pre-immune bleeds from all the mice were collected seven days before the priming with antigen. Mice were immunized sub-cutaneous (SC) with 20μg of antigens, respectively. The recombinant proteins were emulsified with addavax (Invivogen) and montanide ISA 720 (Seppic). They were adsorbed on Alhydrogel (Sigma) on day 0, followed by booster doses with the same formulations on days 14 and 28. The terminal bleeds were collected on day 42. Serum from the immunized mouse groups was tested for antigen-specific antibody titer by enzyme-linked immunosorbent assay (ELISA). Briefly, 200ng of recombinant protein was coated in each well of a 96- well plate (Nunc) and incubated for 12 hrs. at 4°C. Coated plates were blocked with 5% skimmed milk at 37°C for 2 hrs. After blocking, mouse serum was added in a serial dilution starting from 1:100 to 1:1280000 dilution, with each dilution added in duplicate. Plates were incubated for 1 hr. at 37°C, followed by washing with Phosphate buffer saline containing 0.05% Tween 20 (Sigma) and incubation with horseradish peroxidase (HRP) conjugated anti-mouse secondary antibody (Sigma). The assay was developed by adding o-phenylenediamine dihydrochloride (Sigma) and hydrogen peroxide (Sigma), followed by adding 2M sulfuric acid to stop the reaction. Optical density was measured at 492nm. Pre-immune sera and sera from adjuvant control mice were used as controls.

### Survival curve of immunized mice after spore challenge

After the collection of terminal bleeds at day 42, groups of twelve immunized mice were intraperitoneally challenged with 10^4^ virulent spores of *B. anthracis* (35). The virulent encapsulated *B. anthracis* strain (pXO1+ and pXO2+) (16) used for mice challenge is a clinical isolate obtained from an infected carcass at DRDE (Gwalior, MP) and has been demonstrated to be virulent in mice (6, 35). The spores were produced as described (36). Spore-challenged mice were monitored every 24 hrs. for 15 days, and protection provided to the mouse groups by vaccine formulations was assessed by the percentage of mice that survived in each group after 15 days of the challenge. The survival of immunized mouse groups was analyzed by the Kaplan-Meier curve.

### Cytokine profile in the immunized mice sera

Six weeks after primary immunization, the mouse serum was collected from the mouse groups. The pooled serum from all mice in each group was quantitatively analyzed for the various Th1, Th2, and Th17 cytokines. Pooled serum from all the mice immunized with a particular formulation was used for cytokines measurements using Cytometric Bead Assay (CBA) Mouse Th1/Th2 and Th17 Cytokines kit (BD Biosciences), according to the instructions provided by the manufacturer. The data were acquired on BD FACS ARIA (Becton Dickinson) and analyzed using the FCAP Array software V3.0 (Becton Dickinson).

### Statistical analysis

The results are reported as mean ± SE post-data preparation and the statistical analysis using GraphPad Prism. The statistical significance of antibody titer, toxin neutralization assay, and cytokine level data was calculated using one-way ANOVA. The survival curve for the *B. anthracis* spore challenge experiment was evaluated using Kaplan–Meier survival curve (GraphPad Prism, La Jolla, CA, USA). The following denotations highlight statistically significant differences between the groups: * for *P*-value <0.05, ** for *P*-values <0.01, and *** for *P*-values <0.001.

## Results

### Protective antigen subunit antigens were expressed as soluble proteins, and PA-FL was expressed as inclusion bodies

**Figure 1:**
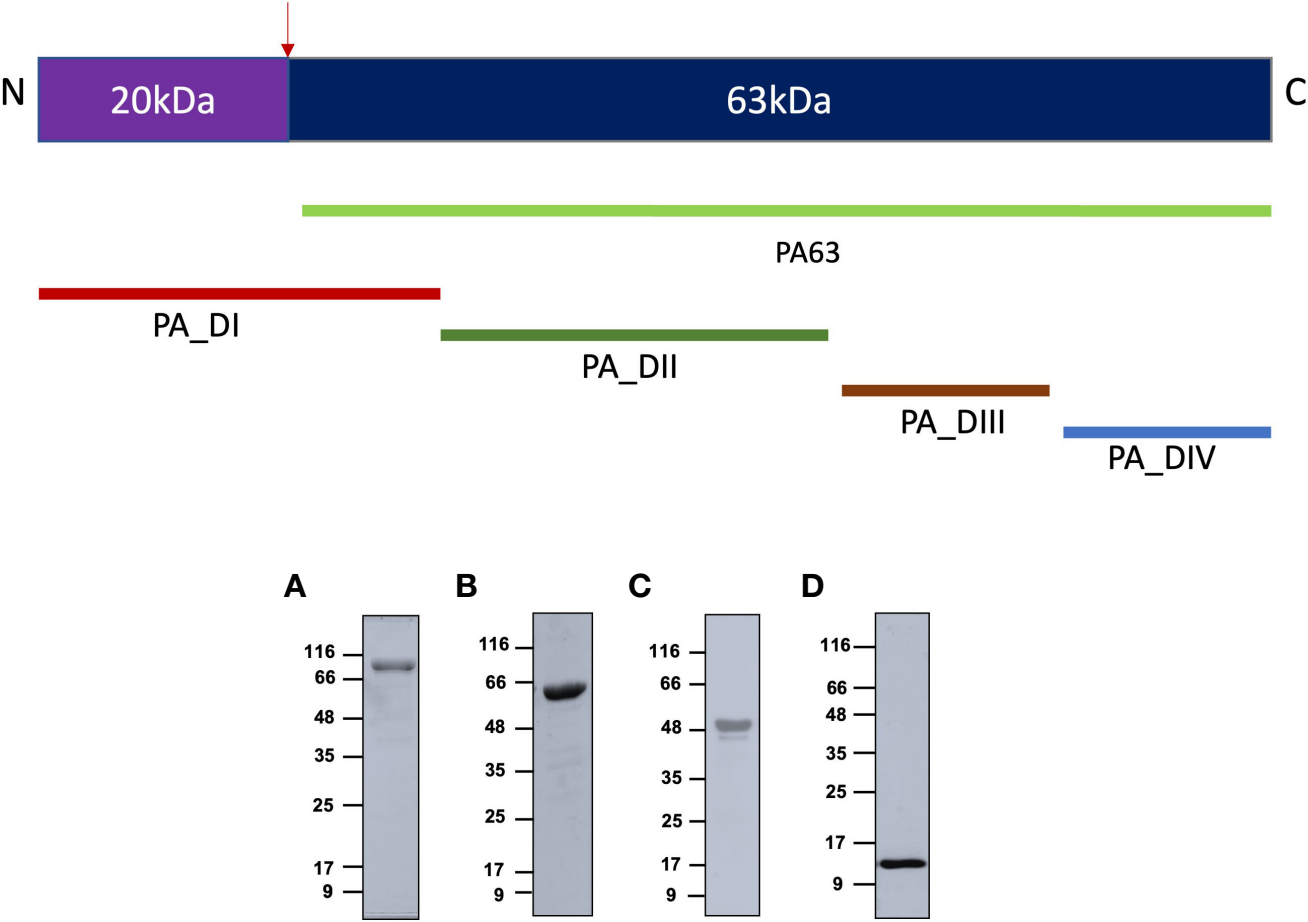
Expression of recombinant antigens in *E coli*: The recombinant protective antigen constructs were produced in *E coli* strains. The four antigens produced were full-length Protective antigen (83 kDa), 63 kDa C-terminal fragment of Protective antigen, fusion construct of Protective antigen domain 1-domain 4 (55 kDa), and domain 4 (15 kDa). The schematic represents all the constructs produced in the study. **(A)** Protective antigen was expressed as inclusion bodies in *E coli* BL21 (DE3) strain. Inclusion bodies were solubilized and refolded in refolding buffer. **(B)** PA63 was co-expressed with Gro-EL/ES molecular chaperones as soluble protein in *E coli* BL21 (DE3) strain. **(C)** PA-D1-4 was expressed as a soluble protein in *E coli* ArcticExpress (DE3) strain. **(D)** PA-D4 was expressed as a soluble protein in *E coli* BL21 (DE3) strain. All the recombinant proteins were purified to homogeneity using various chromatographic methods.

A 735-amino acids sequence of Protective antigen (PA-FL) excluding the signal peptide was produced as a recombinant protein in *E. coli* (UniProt accession number; Q68GS1). All the constructs were expressed with C-terminal 6X-His-tag in *E coli*. The 83 kDa PA-FL protein was expressed in inclusion bodies, which were refolded under denaturing conditions after purification by immobilized metal affinity chromatography and ion-exchange chromatography. Other constructs (PA63, PA-D1-4, and PA-D4) were expressed as soluble proteins. PA63 was expressed in the BL21 (DE3), co-transformed with the GroEL/ES molecular chaperones expressing plasmid (Takara). PA-D1-4 was expressed in *E. coli* ArcticExpress (DE3) cell line, and PA-D4 was expressed in BL21 (DE3). The recombinant proteins were purified to homogeneity by immobilized metal affinity chromatography, ion-exchange chromatography, and size exclusion chromatography (**Figures 1**, **S4**).

### PA-FL and PA-D1-4 are sensitive to trypsin cleavage

Domain 1 of the PA consists of the trypsin-sensitive site (RKKR), and cleavage at this site exposes the binding sites for the lethal factor (LF) and/or edema factor (EF). Physiologically, domain 1 of the protective antigen is cleaved by a host protease, Furin. Trypsin and furin are functionally similar as both cleave the proteins from the C-terminus of Arginine (R) and Lysine (K). The trypsin-sensitive site is present only in domain 1 of PA and the cleavage of PA by trypsin excises a 20 kDa fragment and exposes the functional translocase protein, PA63, for binding the lethal factor (LF) and/or edema factor (EF). A trypsin digestion assay was performed to analyze the trypsin sensitivity of the PA constructs comprising domain 1 (i.e., PA-FL and PA-D1-4). Trypsin treatment is expected to cleave the antigens and excise the 20 kDa fragment from the N-terminus. Protein profiles of the digested proteins post-trypsin treatment were analyzed by SDS-PAGE. The cleaved 20 kDa fragment was detected from the trypsin digestion of both the proteins i.e., PA-FL (83kDa) and PA-D1-4 (55kDa), yielding the respective 63 kDa and 35 kDa fragments (**Figure S2**).

### All the recombinant proteins retain the binding feature to the mammalian cells

**Figure 2:**
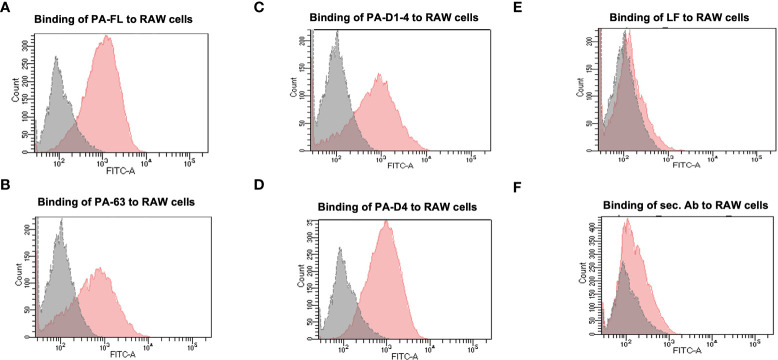
Binding assay to the mammalian cells: The recombinant Protective antigen constructs PA-FL, PA63, PA-D1-4, and PA-D4 produced in *E coli* were analyzed for their functionality. Protective antigen binds to the anthrax toxin receptors on the mammalian cells through PA-D4. All the recombinant antigens have common cell-binding domain 4 and their functionality was assessed by FACS-based cell binding assay. The representative flow cytometry histograms were demonstrated for the binding of recombinant protein i.e., PA-FL **(A)**, PA-63 **(B)**, PA-D1-4 **(C)**, and PA-D4 **(D)** to RAW 264.7 cells. The gray shading represents the RAW 264.7 cells without incubation with recombinant proteins and the red shading represents the RAW 264.7 cells incubated with the recombinant antigens. RAW 264.7 cells were incubated with LF **(E)**, and only secondary antibodies **(F)** were used as negative controls. Each experiment was repeated thrice.

All of the recombinant proteins in our portfolio comprise the host cell binding moiety of PA, i.e., Domain 4. Therefore, we analyzed the cell-binding activity of all recombinant proteins with the murine macrophage cell line, RAW 264.7, in a flow cytometry-based assay. Mammalian cells express the anthrax toxin receptors (TEM8 and CMG2) on their surface (37). Thus, we have assessed the receptor-binding ability of our recombinant antigens. Confluent RAW 264.7 cells were incubated with 5 μg/ml recombinant proteins for 1 hour. Primary anti-PA-FL polyclonal mouse serum followed by anti-mouse Alexa Fluor 488 secondary antibodies were used to detect the binding of recombinant proteins to the RAW 264.7 cells by flow cytometry analysis. All four recombinant proteins in our portfolio exhibited functional cell-binding activity in our FACS-based functional assays. (**Figures 2A** **–D**). The flow cytometry histograms show that the fluorescence peak for all the recombinant antigens is at the same point. However, a higher proportion of the population was found positive for binding with PA-FL and PAD4, which is reflected in the higher cell counts represented in the histogram. The cell-binding activity of the other two constructs (PA63 and PA-D1-4) was relatively lesser than PA-FL and PAD4 (**Figures 2A** **–D**). The recombinant lethal factor (LF) protein, known not to exhibit cell-binding activity was used as a negative control. As expected, the LF protein did not exhibit any cell-binding activity (**Figure 2E**). The RAW 264.7 cells incubated with only secondary antibodies in the absence of the primary anti-PA-D4 antibodies were used as a control for analyzing any non-specific binding of the secondary antibodies with the Fc receptors on the RAW 264.7 cells (**Figure 2F**). Our results with the mammalian cell binding assay demonstrates that all the recombinant antigens exhibit cell-binding activity.

### Antibody generation in small animals and quantitative estimation using ELISA

**Figure 3:**
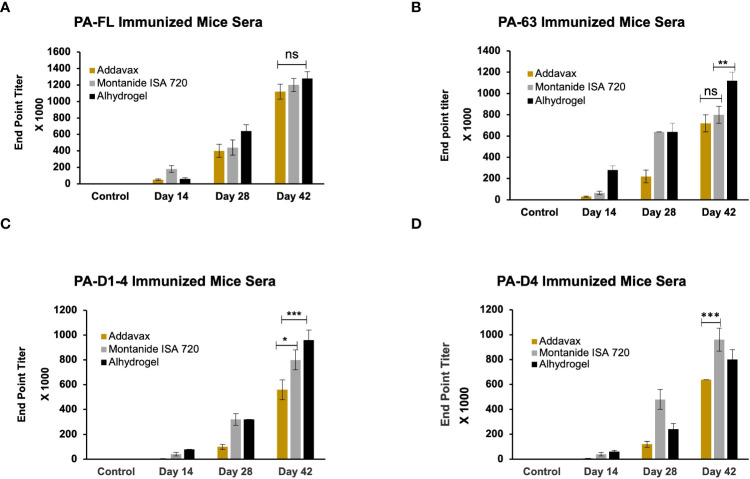
The endpoint titer of the antigen-specific antibody in the serum of immunized animals: The female BALB/c mice were immunized with the formulations of different protective antigen constructs. Four antigens formulated with three adjuvants (i.e., Addavax, Montanide ISA 720, and Alhydrogel) were tested for their antibody titers. Sera from the immunized mice were collected at days 14, 28, and 42 post-primary immunization. Mice sera were serially diluted from 1:100 to 1:1280000. Sera from the unimmunized mice were used to determine the cut-off value for endpoint titers. **(A)** Represents the endpoint titers of antigen-specific antibodies in the sera of mice immunized with PA-FL-based formulations. **(B)** Represents the endpoint titers of antigen-specific antibodies in the sera of mice immunized with PA63-based formulations. **(C)** Represents the endpoint titers of antigen-specific antibodies in the sera of mice immunized with PA-D1-4-based formulations. **(D)** Represents the endpoint titers of antigen-specific antibodies in the sera of mice immunized with PA-D4-based formulations. Statistically significant differences between the groups are highlighted by the following denotations: * for *P*-value <0.05, ** for *P*-values <0.01, and *** for *P*-values <0.001. ns, non-significant.

BALB/c mice were immunized through the subcutaneous route of administration with 20 µg of each antigen. The recombinant proteins were adsorbed on alhydrogel (Sigma) as well as emulsified with addavax (Invivogen), or montanide ISA 720 (Seppic). The control group mice were immunized with each adjuvant alone. Antigen-specific antibody titers for all antigen-adjuvant formulations were measured by indirect ELISA. A group of eight mice was immunized with each vaccine formulation, and endpoint titers were calculated as an average of all mice. The maximum end-point titer of 1.28 x 10^6^ was observed in mice immunized with the PA-FL alhydrogel formulation. The PA-FL montanide formulation elicited anti-PA-FL antibody titers of 1.2 x 10^6^, while the PA-FL addavax formulation induced antibody titers of 1.12x10^6^ (**Figure 3A**). End-point titers of 1.12 x 10^6^ were observed in mice immunized with the PA63 alhydrogel formulation. The PA63-montanide and PA63 addavax formulations elicited the anti-PA63 antibody titers in the range of 8 x 10^5^, and 7.2 x10^5^ respectively (**Figure 3B**). End-point titers of 9.6 x 10^5^ and 8 x 10^5^ were observed in mouse groups immunized with the PA-D1-4 alhydrogel and PA-D1-4 montanide formulations respectively. PAD-1-4-addavax formulation induced antibody titer of 5.6 x10^5^ (**Figure 3C**). The end-point titers of 9.6 x 10^5^, 8 x 10^5^, and 6.4 x10^5^ were observed in the mouse groups immunized with the PA-D4 montanide, PA-D4 alhydrogel, and PA-D4 addavax formulations respectively (**Figure 3D**). The endpoint titer values represent the average of the individual titers of all eight mice in each group.

### PA-FL addavax and PA63 addavax formulations exhibit the highest protection in mice challenged with *B. anthracis* spores

**Figure 4:**
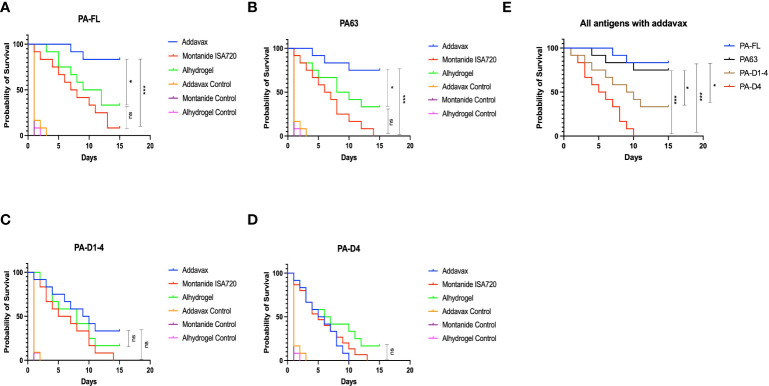
Mouse survival curve. Female BALB/c mice (6–8 weeks) were immunized with Protective Antigen (PA-FL), PA63, PA-D1-4, and PA-D4. Each antigen was formulated with three adjuvants i.e., Addavax, Montanide ISA 720, and Alhydrogel before administration to the mice *via* a subcutaneous route. After collecting terminal bleed, on day 43rd day, mice in each group (*n* = 12) were challenged with 0.5 × 10^3^ virulent spores of *Bacillus anthracis*. Mice were monitored for 15 days for death events in each group. The survival curve was plotted to compare the protection efficiency in vaccinated mice groups over the control placebo mice group. The survival curve for anthrax spore challenge experiments was evaluated using Kaplan–Meier survival estimates (GraphPad Prism, La Jolla, CA, USA). Blue, red, and green shading represent the formulation with Addavax, Montanide ISA 720, and Alhydrogel, respectively. **(A)** Represents the survival curve of the mice groups immunized with PA-FL-based formulations. **(B)** Represents the survival curve of the mice groups immunized with PA63-based formulations. **(C)** Represents the survival curve of the mice groups immunized with PA-D1-4-based formulations. **(D)** Represents the survival curve of the mice groups immunized with PA-D4-based formulations. **(E)** Represents the comparative survival curve of mouse groups immunized with addavax formulations with all four antigens. Statistically significant differences between the groups are highlighted by the following denotations: * for *P*-value <0.05, ** for *P*-values <0.01, and *** for *P*-values <0.001. ns, non-significant.

Mouse groups (twelve mice per group) immunized with various formulations were challenged with a lethal dose of *B. anthracis* spores on day 43 post-immunization. Post-challenge, the survival of the mice in each group was monitored for 15 days, and Kaplan Meier survival curves were plotted to assess the protective efficacy of the vaccine formulations. The mouse group immunized with the PA-FL addavax formulation exhibited the highest survival of 83.3% until 15 days post-challenge (**Figure 4A**). In comparison, the mouse groups immunized with the PA-FL alhydrogel, and PA-FL montanide ISA 720 formulations exhibited the survival rates of 33.3% and 8.33% respectively (**Figure 4A**). The PA63 addavax formulation exhibited the survival of 75%. PA63 alhydrogel formulation exhibited protection in 33.3% of mice while no mice survived till 15 days in PA63 montanide ISA 720 immunized mice. (**Figure 4B**). Respectively, 33.3% and 16.6% of PA-D1-4 addavax and PA-D1-4 alhydrogel immunized mice survived till day 15. However, no mice survived in the PA-D1-4 montanide ISA720 immunized group (**Figures 4C**). PA-D4-based formulations were found to be least effective with no mice survived with addavax and montanide ISA 720 formulations and 16% of mice survived till day 15 with alhydrogel based formulation (**Figure 4D**). Thus, our result demonstrates that PA-FL addavax and PA63 addavax formulations elicit the higher protection in their respective groups (**Figures 4A** **, B**). We analyzed the protective efficacy of all four antigens with the addavax formulations and our result demonstrates that PA-FL addavax and PA63 addavax formulations are similarly efficient in protecting the immunized mice which is significantly better than the PA-D1-4 and PA-D4 based formulations (**Figure 4E**). Interestingly, the addavax based formulations showed lower antibody titers than the other two adjuvant formulations (**Figure 3**) but elicited higher protection, thus suggesting that the addavax based formulation also induced cell-mediated responses. Addavax induces both Th1 and Th2 responses (**Figure 6**), which could be attributed to the activation of both humoral and cell-mediated immunity, eventually leading to higher protection in challenged mice. Overall, the result of our mouse challenge study demonstrates that the PA-FL addavax and PA63 addavax formulations elicit significantly higher protective efficacy than the alhydrogel and montanide based formulations of these antigens respectively (**Figures 4A, B**). Secondly, the PA-FL and PA63 formulations with addavax elicit the equivalent protection in spore-challenged mice.

### PA-FL addavax, PA-FL alhydrogel, PA63 addavax, and PA63 alhydrogel formulations exhibited equivalent toxin-neutralizing activity

**Figure 5:**
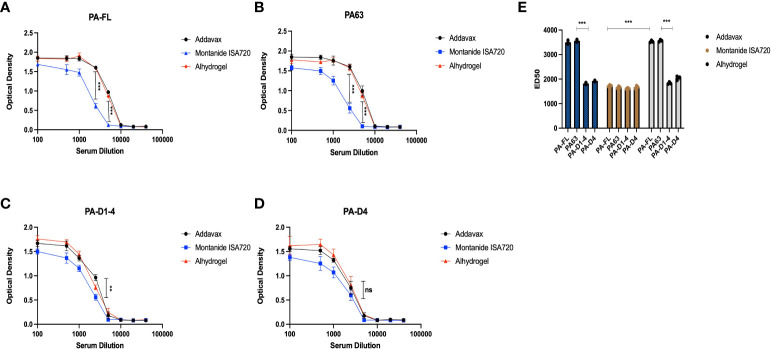
Toxin neutralization assay: The pooled sera from the mice immunized with different formulations were analyzed for their efficacy in neutralizing the anthrax lethal toxin. The different dilutions of mice sera were mixed with lethal doses of both protective antigen (1 μg/ml) and lethal factor (1.5 μg/ml) and incubated for 1 hr. The sera and recombinant toxin components were added to the RAW 264.7 cells, and the lethal effect of anthrax lethal toxin was analyzed. **(A)** Represents the toxin-neutralizing efficacy of mice sera immunized with PA-FL-based formulations. **(B)** Represents the toxin-neutralizing efficacy of mice sera immunized with PA63-based formulations. **(C)** Represents the toxin-neutralizing efficacy of mice sera immunized with PA-D1-4-based formulations. **(D)** Represents the toxin-neutralizing efficacy of mice sera immunized with PA-D4-based formulations. **(E)** Represent the ED_50_ values for the mice sera collected from different mice groups. Statistically significant differences between the groups are highlighted by the following denotations: * for *P*-value <0.05, ** for *P*-values <0.01, and *** for *P*-values <0.001. ns, non-significant.

To evaluate the levels of protective immune responses that could explain the results of the mice challenge studies, we analyzed the efficacy of the mice sera in *in-vitro* toxin neutralization assays (TNA). The assay was standardized by titrating the toxin concentration for cytotoxicity activities of the recombinant protective antigen (PA-FL) and lethal factor (LF) (**Figure S3**). Recombinant PA-FL and LF were able to induce cytotoxicity in RAW264.7 (**Figure 5**). However, we identified the minimal concentrations of PA-FL and LF that produce 90% cytotoxicity for assessing the toxin-neutralizing efficiency of mice sera in TNA. MTT-based cell viability assays were performed using a range of increasing concentrations of either PA-FL or LF at a fixed concentration of the other. Our result demonstrates that the minimum inhibitory concentrations of PA-FL and LF required for inducing 90% cytotoxicity were 1 µg/ml and 1.5 µg/ml, respectively (**Figure S3**). The minimum effective concentrations of PA-FL and LF were mixed with increasing dilutions of pooled mouse serum (1:100 – 1:40000) before incubation with the RAW264.7 cells. The toxin-serum mixtures were added to RAW264.7 cells at a confluence of 90% in 96 well plates. The toxin-neutralization efficacy of antibodies was analyzed by the MTT-based cell viability assays. Mouse antibodies raised against the PA-FL addavax, PA-FL alhydrogel, PA63 addavax, and PA63 alhydrogel formulations exhibited the equivalent toxin-neutralizing activity leading to the inhibition of cell death (**Figures 5A** **, B**). In contrast, the montanide ISA 720 formulations with PA-FL and PA63 elicited antibodies exhibited lower efficacy in the TNA assays (**Figures 5A** **, B**). Sera from mouse groups immunized with PA-D1-4 and PA-D4 formulations were found to be less effective in neutralizing recombinant lethal toxin (**Figures 5C, D**). Toxin neutralization is measured by observing the cell viability, which would decrease due to the toxin effect and be protected by the toxin neutralization by the respective serum. ED_50_ is the effective dilution of the pooled sera that neutralizes the toxin activity by 50%. To calculate the ED_50_, the corresponding OD_50_ value representing 50% toxin neutralization is first calculated by the formula (= O.D. _max_ - O.D. _min)/_2). The dilution of the mice sera corresponding to OD_50_ was analyzed using the graphical plot equations (**Figure 5E**).

### PA-addavax immunized mice serum has higher levels of Th1, Th2, and Th17 cytokines

**Figure 6:**
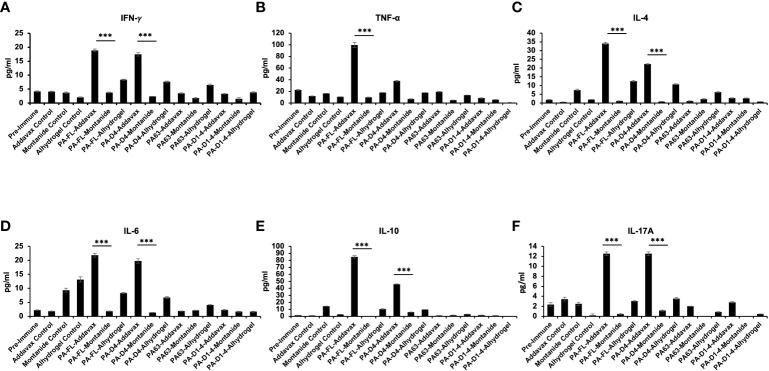
Cytokine release profile in the immunized mice sera: The pooled sera from all the immunized mouse groups were quantitatively analyzed for the different Th1, Th2, and Th17 cytokines using cytometric bead assay. **(A, B)** Represents the concentration (pg/ml) of Th1 cytokines TNF-α and IFN-γ in the serum of mice immunized with all the antigen-adjuvants formulations. **(C–E)** Represents the concentration (pg/ml) of Th2 cytokines IL-4, IL-6, and IL-10 in the serum of mice immunized with all the antigen-adjuvants formulations. **(F)** Represents the concentration (pg/ml) of Th17 cytokine IL17A in the serum of mice immunized with all the antigen-adjuvants formulations. Pre-immune mice sera and adjuvant control mice sera were used as negative controls. Statistically significant differences between the groups are highlighted by the following denotations: * for *P*-value <0.05, ** for *P*-values <0.01, and *** for *P*-values <0.001.

The Th1, Th2, and Th17 cytokines levels in the sera from the immunized mice were quantitatively analyzed using a cytometric bead assay. The highest levels of both Th1 cytokines (IFN-γ and TNF-α) were observed in PA-FL addavax formulation (average concentration of IFN-γ-18.9 pg/ml and TNF-α- 99.6 pg/ml) followed by PA-D4 addavax formulations (average concentration of IFN-γ-17.5 pg/ml and TNF-α- 37.88 pg/ml) (**Figures 6** **A, B**). Thus, the recombinant PA-FL addavax formulation was the most efficient in inducing the release of both Th1 cytokines. For Th2 cytokines (IL-4, IL-6, and IL-10), the highest levels were detected in PA-FL addavax formulations (average concentration of IL-4 -34.09 pg/ml, IL-6- 21.84 pg/ml and IL-10- 84.95pg/ml) followed by PA-D4 addavax formulations (average concentration of IL-4 -22.29 pg/ml, IL-6 - 19.76 pg/ml and IL-10- 45.95 pg/ml) (**Figures 6C** **–E**). PA-FL alhydrogel formulation induced the release of only Th2 cytokines, IL-4 (12.58 pg/ml), and IL-6 (8.36 pg/ml) (**Figures 6C, D**). No significant cytokines production was observed in mice immunized with montanide ISA 720 formulations. The highest concentration of Th17 cytokine, IL-17A, was observed in mice immunized with PA-FL addavax and PA-D4-Addavax (12.52 pg/ml each) (**Figure 6F**). Overall, the Th1, Th2, and Th17 cytokine release profiles of the pooled sera of the PA-FL addavax immunized mice is significantly higher than the PA-FL montanide ISA 720 and PA-FL alhydrogel immunized mice sera. Comparing the different antigens with addavax formulation, PA-D4 was shown to induce the release of Th1, Th2, and Th17 cytokines in mice sera (**Figure 6**). This result may correlate with the results from the mice spore challenge experiment (**Figure 4**).

## Discussion

*Bacillus anthracis* is one of the most virulent and lethal bacterial pathogens. *B. anthracis* infection has an incubation period of 1-7 days and antibiotic therapies are effective only in the early phase of infection (38–40). Early diagnosis of *B. anthracis* infection is difficult due to the onset of mild and inconsistent symptoms, and the early phase is followed by intensive toxemia which is very difficult to control at the later stages. Therefore, it is imperative to develop novel anthrax intervention therapeutics that work at very early stages or before the establishment of infection. The currently available anthrax vaccines are either whole-cell vaccines based on attenuated spores or acellular vaccines such as AVA (Anthrax Vaccine Adsorbed) composed of the *B. anthracis* culture supernatant containing secreted anthrax exotoxins (41). AVA is the only FDA-approved vaccine produced by Emergent BioSolutions (Rockville, Maryland, USA) under the trade name BioThrax. This vaccine is used for both pre-exposure and post-exposure prophylaxis (42). Both AVA and attenuated spores-based vaccines face the major limitations of either reversion to virulent forms (attenuated spore-based vaccine) or the risk of the unregulated higher concentration of exotoxins that may produce significant reactogenicity in some patients (AVA). Secondly, AVA has a short persistence time and thus requires several booster doses (generally five doses in the first 18 months and an annual dose afterward) that have their challenges (43). Therefore, there is a clear need to develop the next generation of safe and efficient anthrax vaccines that can overcome the limitations of vaccine intoxication and prolong its efficacy. In this regard, it is widely accepted that recombinant protein-based vaccines could fulfill such a role. Thus, efforts to produce recombinant anthrax toxin proteins and analysis of their immune potential are essential. Translationally, it is always easy and economical to produce the recombinant antigens which are smaller and are expressed as soluble proteins in *E. coli*. Therefore, in the present study, we aimed to produce the subunit constructs of PA as soluble proteins and analyze their vaccine potential with already approved human-compatible adjuvants.

Protective antigen (PA) is the toxin-translocating component of *B. anthracis* A-B toxin and has been implicated as an efficient vaccine candidate (26). In a mouse challenge study, immunization with recombinant PA was reported to elicit protection against *B. anthracis* spores (44). PA is a highly immunogenic protein comprising several B-cell and T-cell epitopes (45). The major challenge with a subunit PA-based anthrax vaccine is the poor stability and thermolabile characteristic of full-length PA recombinant protein (32, 33). Besides that, in earlier studies, the recombinant PA has been produced as inclusion bodies in *E. coli* and it is very difficult and uneconomical to translate that process to the industrial scale. Thus, our study is focused on primarily two objectives; to produce soluble and immunogenic versions of recombinant PA constructs that elicit optimal protection in mice challenge studies. Secondly, analyze the vaccine potential of our antigens in combination with human-compatible adjuvants.

In the present study, we optimized the expression of all the recombinant proteins of our portfolio in a soluble form so that their structural conformation mimics that of the *B. anthracis* native protein and it is very convenient for them to purify to homogeneity. We successfully produced all the recombinant proteins in a soluble form except for the full-length PA, which was expressed as inclusion bodies. The recombinant antigens were functionally characterized for their cell binding activity and trypsin sensitivity. All our recombinant antigens were found to bind the murine macrophage cell line. Native PA has a trypsin-sensitive site RKKR in domain 1 and is known to be cleaved by membrane-bound furin, which excises an N-terminal 20 kDa fragment from PA, exposing its binding site with either LF or EF (19). Domain 1 containing recombinant proteins i.e., PA-FL, and PA-D1-4, were treated with trypsin and observed to yield the N-terminal 20 kDa fragment suggesting that the recombinant proteins were expressed in a functional conformation that mimics the native PA protein structure in *B. anthracis*.

PA binds with the host cell receptors and acts as a translocase. Thus, we assessed the translocase activity of recombinant PA-FL with the mammalian cells. The combination of recombinant PA-FL and LF (35) efficiently induced cell death, suggesting that both recombinant proteins are functionally active. PA63 heptamer is involved in the formation of a pre-pore and facilitates toxin translocation into the cells (46). However, the combination of our recombinant PA63 and LF failed to cause cell death, suggesting that the PA63 could not translocate LF into the cell, which could be due to the non-functional conformation of the LF/EF binding sites and/or oligomerization region on PA63.

In mice challenge studies, we validated the vaccine potential of our recombinant antigens formulated with human-compatible adjuvants i.e., Alhydrogel, Montanide ISA 720, and Addavax. BALB/c mice were immunized with the respective antigen-adjuvant formulations, and the protective efficacy was assessed by challenging the mouse groups with a lethal dose of *B. anthracis* virulent spores (35). Mouse groups immunized with PA-FL addavax and PA63 addavax formulation elicit higher protection in our spore challenge studies. Further assessment of the antibody titers and their toxin-neutralizing efficacy of mice sera suggested that while the total antibody responses elicited by the addavax formulation was the least, they have the similar toxin-neutralizing efficacy comparable to that of the alhydrogel formulations. Immunization with PA-FL addavax and PA63 addavax formulations provided significantly higher protection than the PA-FL montanide ISA 720 and PA63 montanide ISA 720 formulations respectively. The mice immunized with PA-FL alhydrogel and PA63 alhydrogel showed higher survival than PA-FL montanide ISA 720 and PA63 montanide ISA 720 respectively, however, the difference was not significant. PA-D1-4 and PA-D4 formulations with all three adjuvants did not demonstrate any survival difference. A comparison of the survival data from all four antigens with addavax formulation demonstrates that PA-FL and PA63 provide similar protection, which is significantly better than PA-D1-4 and PA-D4.

Addavax activates the balanced Th1 and Th2 responses and activates both humoral and cell-mediated immunity (47, 48). Alhydrogel is primarily the activator of the Th2 response while Montanide ISA 720 activates the innate inflammatory response and recruits antigens-presenting cells at the site of injection (49) We have analyzed the level of Th1, Th2, and Th17 cytokines in the pooled serum of immunized mice and found the highest level of all the cytokines in the mice sera immunized with PA-FL-Addavax formulation. These increased levels of Th1 and Th2 cytokines represent the multi-dimensional activation of immune response that could correlate to higher protection in the challenged mice.

In conclusion, our study has demonstrated that the PA-FL addavax and PA63 addavax formulations have equivalent efficacy in providing protection to the immunized mice and generating toxin-neutralizing antibodies which are demonstrated by both *in-vivo* and *in-vitro* experiments. The PA-FL is produced as inclusion bodies in *E. coli* while PA63 was expressed as a soluble protein with the co-expression of molecular chaperons GroEL/ES. Considering the equivalent efficacy of both the antigens i.e., PA-FL and PA63 as vaccine candidates, the relative ease of production of recombinant PA63 in *E. coli* has considerable translational relevance for the development of an efficient next-generation anti-toxin vaccine against anthrax (50).

## Data availability statement

The datasets presented in this study can be found in online repositories. The names of the repository/repositories and accession number(s) can be found: https://www.uniprot.org/uniprotkb/Q68GS1/entry.

## Ethics statement

The animal study was reviewed and approved by Institutional Animal Ethics Committee (Jawaharlal Nehru University) and Council for the Purpose of Control and Supervision of Experiments on Animals (CPCSEA, Ministry of Social Justice and Empowerment, Government of India).

## Author contributions

SS and DG designed the study. SS, VB, GS, and RS performed the experiments. DG supervised the study. RB provided key reagents and facilities. SS and DG analyzed the results. SS and DG wrote the manuscript and approved it by all authors. All authors contributed to the article and approved the submitted version.

## References

[1] Moayeri M Leppla SH Vrentas C Pomerantsev AP Liu S. Anthrax pathogenesis. Annu Rev Microbiol (2015) 69:185–208. doi: 10.1146/annurev-micro-091014-104523 26195305

[2] Moir A Cooper G. Spore germination. Microbiol Spectr (2015) 3(6). doi: 10.1128/microbiolspec.TBS-0014-2012 27337279

[3] Goel AK. Anthrax: A disease of biowarfare and public health importance. World J Clin cases (2015) 3(1):20–33. doi: 10.12998/wjcc.v3.i1.20 25610847 PMC4295216

[4] Jernigan JA Stephens DS Ashford DA Omenaca C Topiel MS Galbraith M. Anthrax bioterrorism investigation team. bioterrorism-related inhalational anthrax: the first 10 cases reported in the united states. Emerging Infect Dis J (2001) 7(6):933–44. doi: 10.3201/eid0706.010604 PMC2631903 11747719

[5] Binkley CE Cinti S Simeone DM Colletti LM. Bacillus anthracis as an agent of bioterrorism: a review emphasizing surgical treatment. Ann Surg (2002) 236(1):9–16. doi: 10.1097/00000658-200207000-00004 12131080 PMC1422543

[6] Sharma S Bhatnagar R Gaur D. Bacillus anthracis poly-γ -D-Glutamate capsule inhibits opsonic phagocytosis by impeding complement activation. Front Immunol (2020) 11:462. doi: 10.3389/fimmu.2020.00462 32296419 PMC7138205

[7] Sharma S Bhatnagar R and Gaur D. Complement evasion strategies of human pathogenic bacteria. Indian J Microbiol (2020) 60(3):283–96. doi: 10.1007/s12088-020-00872-9 PMC7329968 32655196

[8] Chabot DJ Ribot WJ Joyce J Cook J Hepler R Nahas D. Protection of rhesus macaques against inhalational anthrax with a bacillus anthracis capsule conjugate vaccine. Vaccine (2016) 34(34):4012–6. doi: 10.1016/j.vaccine.2016.06.031 27329184

[9] Chabot DJ Joyce J Caulfield M Cook J Hepler R Wang S. Efficacy of a capsule conjugate vaccine against inhalational anthrax in rabbits and monkeys. Vaccine (2012) 30(5):846–52. doi: 10.1016/j.vaccine.2011.12.010 22172509

[10] Collier RJ. Membrane translocation by anthrax toxin. Mol Aspects Med (2009) 30(6):413–22. doi: 10.1016/j.mam.2009.06.003 PMC2783560 19563824

[11] Young JA Collier RJ. Anthrax toxin: receptor binding, internalization, pore formation, and translocation. Annu Rev Biochem (2007) 76:243–65. doi: 10.1146/annurev.biochem.75.103004.142728 17335404

[12] Cote CK Welkos SL Bozue J. Key aspects of the molecular and cellular basis of inhalational anthrax. Microbes Infect (2011) 13(14-15):1146–55. doi: 10.1016/j.micinf.2011.07.005 21816231

[13] Vitale G Bernardi L Napolitani G Mock M Montecucco C. Susceptibility of mitogen-activated protein kinase kinase family members to proteolysis by anthrax lethal factor. Biochem J (2000) 352(3):739–45. doi: 10.1042/bj3520739 PMC1221512 11104681

[14] Pellizzari R Guidi-Rontani C Vitale G Mock M Montecucco C. Anthrax lethal factor cleaves MKK3 in macrophages and inhibits the LPS/IFN gamma-induced release of NO and TNF alpha. FEBS Lett (1999) 462(1-2):199–204. doi: 10.1016/s0014-5793(99)01502-1 10580119

[15] Lowe DE Glomski IJ. Cellular and physiological effects of anthrax exotoxin and its relevance to disease. Front Cell Infect Microbiol (2012) 2:76. doi: 10.3389/fcimb.2012.00076 22919667 PMC3417473

[16] Liu S Moayeri M Leppla SH. Anthrax lethal and edema toxins in anthrax pathogenesis. Trends Microbiol (2014) 22(6):317–25. doi: 10.1016/j.tim.2014.02.012 PMC4041834 24684968

[17] Friebe S van der Goot FG Bürgi J. The ins and outs of anthrax toxin. Toxins (Basel) (2016) 8(3):69. doi: 10.3390/toxins8030069 26978402 PMC4810214

[18] Hammamieh R Ribot WJ Abshire TG Jett M Ezzell J. Activity of the bacillus anthracis 20 kDa protective antigen component. BMC Infect Dis (2008) 8:124. doi: 10.1186/1471-2334-8-124 18808698 PMC2564935

[19] Petosa C Collier RJ Klimpel KR Leppla SH Liddington RC. Crystal structure of the anthrax toxin protective antigen. Nature (1997) 385(6619):833–8. doi: 10.1038/385833a0 9039918

[20] Feld GK Kintzer AF Tang II Thoren KL Krantz BA. Domain flexibility modulates the heterogeneous assembly mechanism of anthrax toxin protective antigen. J Mol Biol (2012) 415(1):159–74. doi: 10.1016/j.jmb.2011.10.035 PMC3249527 22063095

[21] Hardenbrook NJ Liu S Zhou K Ghosal K Zhou ZH Krantz BA. Atomic structures of anthrax toxin protective antigen channels bound to partially unfolded lethal and edema factors. Nat Commun (2020) 11:840. doi: 10.1038/s41467-020-14658-6 32047164 PMC7012834

[22] Friebe S Deuquet J van der Goot FG. Differential dependence on n-glycosylation of anthrax toxin receptors CMG2 and TEM8. PloS One (2015) 10(3):1–18. doi: 10.1371/journal.pone.0119864 PMC4363784 25781883

[23] Pitt ML Little SF Ivins BE Fellows P Barth J Hewetson J. *In vitro* correlate of immunity in a rabbit model of inhalational anthrax. Vaccine (2001) 19(32):4768–73. doi: 10.1016/s0264-410x(01)00234-1 11535328

[24] Weiss S Kobiler D Levy H Marcus H Pass A Rothschild N. Immunological correlates for protection against intranasal challenge of bacillus anthracis spores conferred by a protective antigen-based vaccine in rabbits. Infect Immun (2006) 74(1):394–8. doi: 10.1128/IAI.74.1.394-398.2006 PMC1346658 16368995

[25] Hopkins RJ Howard C Hunter-Stitt E Kaptur PE Pleune B Muse D. Phase 3 trial evaluating the immunogenicity and safety of a three-dose BioThrax® regimen for post-exposure prophylaxis in healthy adults. Vaccine (2014) 32(19):2217–24. doi: 10.1016/j.vaccine.2014.01.073 24613523

[26] Little SF Ivins BE Fellows PF Pitt ML Norris SL Andrews GP. Defining a serological correlate of protection in rabbits for a recombinant anthrax vaccine. Vaccine (2004) 22(3-4):422–30. doi: 10.1016/j.vaccine.2003.07.004 14670324

[27] Baillie LW Huwar TB Moore S Mellado-Sanchez G Rodriguez L Neeson BN. An anthrax subunit vaccine candidate based on protective regions of bacillus anthracis protective antigen and lethal factor. Vaccine (2010) 28(41):6740–8. doi: 10.1016/j.vaccine.2010.07.075 PMC3008506 20691267

[28] WHO. Anthrax vaccines to humans. Geneva: World Health Organization (2012).

[29] Hugh Dyson E Simpson AJH Gwyther RJ Cuthbertson H Patient DH Matheson M. Serological responses to anthrax vaccine precipitated (AVP) increase with time interval between booster doses. Vaccine (2022) 40(42):6163–78. doi: 10.1016/j.vaccine.2022.08.052 36153153

[30] Gorse GJ Keitel W Keyserling H. Immunogenicity, and tolerance of ascending doses of a recombinant protective antigen (rPA102) anthrax vaccine: a randomized, double blinded, controlled, multicenter trial. Vaccine (2006) 24(33-34):5950–9. doi: 10.1016/j.vaccine.2006.05.044 16797805

[31] Leffel EK Bourdage JS Williamson ED Duchars MG. Recombinant protective antigen anthrax vaccine improves survival when administered as a postexposure prophylaxis countermeasure with antibiotic in the new Zealand white rabbit model of inhalation anthrax. Clin Vaccine Immunol (2012) 19(8):1158–64. doi: 10.1128/CVI.00240-12 PMC3416090 22695155

[32] Radha C Salotra P Bhat R Bhatnagar R. Thermostabilization of protective antigen–the binding component of anthrax lethal toxin. J Biotechnol (1996) 50(2-3):235–42. doi: 10.1016/0168-1656(96)01569-6 8987626

[33] Singh S Ahuja N Chauhan V Rajasekaran E Mohsin Waheed S Bhat R. Gln277 and Phe554 residues are involved in thermal inactivation of protective antigen of bacillus anthracis. Biochem Biophys Res Commun (2002) 296(5):1058–62. doi: 10.1016/s0006-291x(02)02049-1 12207879

[34] Ko EJ Kang SM. Immunology, and efficacy of MF59-adjuvanted vaccines. Hum Vaccin Immunother (2018) 14(12):3041–5. doi: 10.1080/21645515.2018.1495301 PMC6343625 30015572

[35] Aggarwal S Somani VK Gupta S Garg R Bhatnagar R. Development of a novel multiepitope chimeric vaccine against anthrax. Med Microbiol Immunol (2019) 208(2):185–95. doi: 10.1007/s00430-019-00577-x 30671633

[36] Liu H Bergman NH Thomason B Shallom S Hazen A Crossno J. Formation and composition of the bacillus anthracis endospore. J Bacteriol (2004) 186(1):164–78. doi: 10.1128/JB.186.1.164-178.2004 PMC303457 14679236

[37] Merritt C Chun EM Fattah RJ Silva LM Ma QQ Moayeri M. Imaging of anthrax intoxication in mice reveals shared and individual functions of surface receptors CMG-2 and TEM-8 in cellular toxin entry. J Biol Chem (2022) 298(1):101467. doi: 10.1016/j.jbc.2021.101467 34871548 PMC8716333

[38] Head BM Alfa M Sitar DS Rubinstein E Meyers AF. *In vitro* evaluation of the effect of linezolid and levofloxacin on bacillus anthracis toxin production, spore formation and cell growth. J Antimicrob Chemother (2017) 72(2):417–20. doi: 10.1093/jac/dkw427 27798209

[39] Head BM Rubinstein E Meyers AF. Alternative pre-approved and novel therapies for the treatment of anthrax. BMC Infect Dis (2016) 16(1):621. doi: 10.1186/s12879-016-1951-y 27809794 PMC5094018

[40] Cavallo JD Ramisse F Girardet M Vaissaire J Mock M Hernandez E. Antibiotic susceptibilities of 96 isolates of bacillus anthracis isolated in France between 1994 and 2000. Antimicrob Agents Chemother (2002) 46(7):2307–9. doi: 10.1128/AAC.46.7.2307-2309.2002 PMC127281 12069996

[41] Wright JG Plikaytis BD Rose CE Parker SD Babcock J Keitel W. Effect of reduced dose schedules and intramuscular injection of anthrax vaccine adsorbed on immunological response and safety profile: a randomized trial. Vaccine (2014) 32(8):1019–28. doi: 10.1016/j.vaccine.2013.10.039 PMC9067390 24373307

[42] Longstreth J Skiadopoulos MH Hopkins RJ. Licensure strategy for pre- and post-exposure prophylaxis of biothrax vaccine: the first vaccine licensed using the FDA animal rule. Expert Rev Vaccines (2016) 15(12):1467–79. doi: 10.1080/14760584.2016.1254556 27792416

[43] Wolfe DN Espeland EM Gao Y Lu D Blatner G Amass K. Evaluation of BioThrax® and AV7909 anthrax vaccines in adults 66 years of age or older. Vaccine (2020) 38(50):7970–6. doi: 10.1016/j.vaccine.2020.10.053 33129609

[44] Ivins BE Pitt ML Fellows PF Farchaus JW Benner GE Waag DM. Comparative efficacy of experimental anthrax vaccine candidates against inhalation anthrax in rhesus macaques. Vaccine (1998) 16(11-12):1141–8. doi: 10.1016/s0264-410x(98)80112-6 9682372

[45] Abboud N De Jesus M Nakouzi A Cordero RJ Pujato M Fiser A. Identification of linear epitopes in bacillus anthracis protective antigen bound by neutralizing antibodies. J Biol Chem (2009) 284(37):25077–86. doi: 10.1074/jbc.M109.022061 PMC2757211 19617628

[46] Milne JC Furlong D Hanna PC Wall JS Collier RJ. Anthrax protective antigen forms oligomers during intoxication of mammalian cells. J Biol Chem (1994) 269(32):20607–12. doi: 10.1016/S0021-9258(17)32036-7 8051159

[47] Calabro S Tritto E Pezzotti A Taccone M Muzzi A Bertholet S Gregorio ED. The adjuvant effect of MF59 is due to the oil-in-water emulsion formulation, none of the individual components induce a comparable adjuvant effect. Vaccine (2013) 31(33):3363–9. doi: 10.1016/j.vaccine.2013.05.007 23684834

[48] Shi S Zhu H Xia X Liang Z Ma X Sun B. Vaccine adjuvants: Understanding the structure and mechanism of adjuvanticity. Vaccine (2019) 37(24):3167–78. doi: 10.1016/j.vaccine.2019.04.055 31047671

[49] Marques RF de Melo FM Novais JTenório Soares Irene S Bargieri DY Gimenez AM. Immune system modulation by the adjuvants poly (I:C) and montanide ISA 720. Front Immunol (2022) 13:910022. doi: 10.3389/fimmu.2022.910022 35844531 PMC9278660

[50] Bhatwa A Wang W Hassan YI Abraham N Li X-Z Zhou T. Challenges associated with the formation of recombinant protein inclusion bodies in escherichia coli and strategies to address them for industrial applications. Front Bioengineering Biotechnol (2021) 9:630551. doi: 10.3389/fbioe.2021.630551 PMC7902521 33644021

