# Supplementary material for: Recombinant full-length Bacillus Anthracis protective antigen and its 63 kDa form elicits protective response in formulation with addavax
Source: Front Immunol. 2023 Jan 12;13:1075662. doi: 10.3389/fimmu.2022.1075662 (PMC9877290; doi:10.3389/fimmu.2022.1075662)
Supplement: Supplementary file 1 [file DataSheet_1.pdf]

Supplementary Material

Recombinant Full-Length *Bacillus Anthracis* Protective Antigen and Its 63kDa Form Elicits Protective Response in Formulation with Addavax

Shikhar Sharma*, Vanndita Bahl, Gaurav Srivastava, Risha Shamim, Rakesh Bhatnagar, Deepak Gaur

*Corresponding Author

Please address correspondence at the following address:

Shikhar Sharma, Ph.D.
Oncology Science,
University of Oklahoma Health Science Center,
Oklahoma City, Oklahoma, USA
Tel: +1-402-590-4878
E-mail: shikhar.sharma@yahoo.com, shikhar-sharma@ouhsc.edu

Table S1: Primers sequence for production of recombinant proteins

Primer	Sequence
Forward PA-FL	ATTCGGGAATTCCATATGGAAGTTAAACAGGAGAAC
Reverse PA-FL	TATCCGCTCGAGTCCTATCTCATAGCCTTTTTTAG
Forward PA-63	ATTCGGGAATTCCATATGACAAGTGCTGGACCTACGG
Reverse PA-DI	TATAGGATCCATAAGCTGCCACAAGGGG
Forward PA-DIV (BamH1)	ACCTGGATCCCATTATGATAGAAATAACATAGC
Forward PA-DIV (NdeI)	ATTCGGGAATTCCATATGCATTATGATAGAAATAACATAG

Figure S1: Molecular Cloning for the production of recombinant proteins

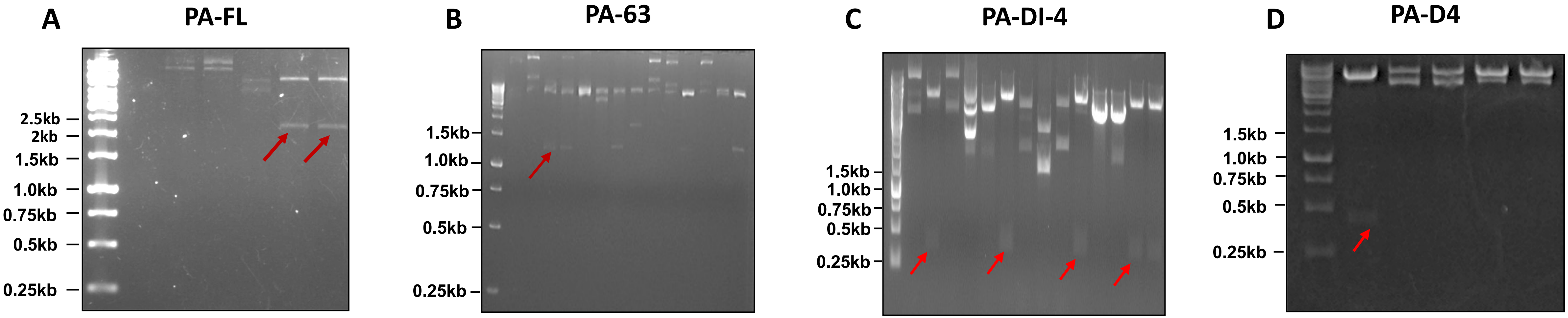

Figure S1: Molecular cloning of PA and sub-constructs genes in pET24b vector: Specific genes of insert were amplified using PA clone in pQE30 vector. Amplified genes and pET24b plasmid were cleaved by restriction enzymes. Cleaved genes and pET24b vectors were ligated and transformed into *E. coli* DH5α cells. Bacterial colonies were screened for the positive clones by restriction digestion of the isolated plasmids. **(A)** 2.3 kb insert was observed in positive clones of full-length PA. **(B)** 1.7 kb insert was observed in positive clones of PA-63. **(C)** PA-DI and PA-D4 was sequentially cloned in pET24b between NdeI/BamHI and BamHI/ XhoI respectively. Plasmid isolated from the transformed colonies were digested with restriction enzymes BamHI and XhoI to confirm the 0.42 kb PA-D4 fall-out. **(D)** 0.42 kb insert was observed in positive clones of PA-D4.

Table S2: Conditions for the production of recombinant antigens

**Protein**	***E. coli* strain**	**Expression**	**Temperature**	**Incubation time (hrs.)**	**IPTG Concentration**
PA-D4	BL21 (DE3)	Soluble	16°C	14	1 mM
PA-63	BL21 (DE3) co-transformed with pGro7	Soluble	16°C	14	1 mM
PA-D1-4	ArcticExpress (DE3)	Soluble	16°C	14	1 mM
PA-FL	BL21 (DE3)	Inclusion bodies	37°C	4	1 mM

Figure S2: Trypsin digestion assay of PA-FL and PA-D1-4

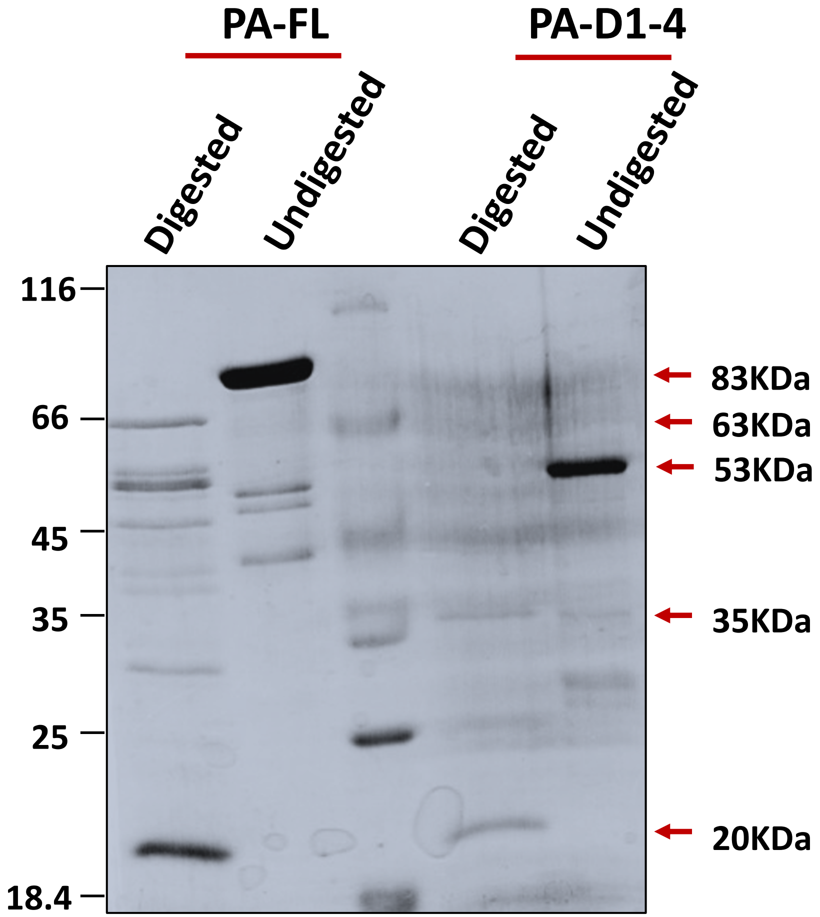

Figure S2: Trypsin Digestion assay: The protective antigen domain I has the trypsin sensitive site (RKKR), which is cleaved by host furin and expose the LF/EF binding site on PA. The cleavage of PA releases a 20 kDa fragment from the N-terminus. The 83 kDa recombinant protective antigen was digested with trypsin at room temperature and run on SDS-PAGE (Lane 1 from left). Prominent 20 kDa and 63 kDa bands were visible. Lane 2 represents the undigested recombinant PA. Another protein tested for trypsin sensitive site is PA-D1-4. Recombinant PAD1-4 was digested with trypsin and resolved on SDS-PAGE. Lane 3 and 4 represent the digested and undigested PA-D1-4 protein respectively. Both the recombinant proteins having domain 1 sequence were found to be having functional trypsin sensitive site.

Figure S3: Optimization of lethal dose of recombinant protective antigen and lethal factor

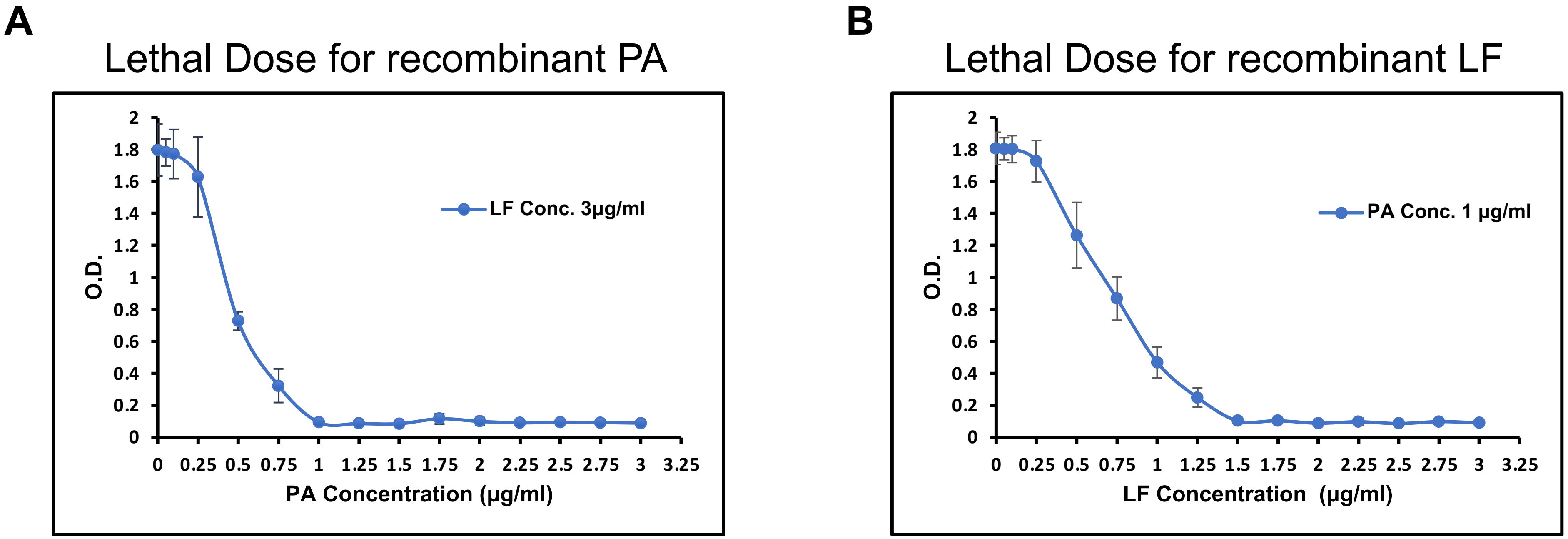

Figure S3: Optimization of lethal dose of recombinant protective antigen and lethal factor: RAW 264.7 cells survival assays were performed for the determination of minimum lethal concentration of recombinant Protective antigen and Lethal factor to kill 90% of the RAW 264.7 cells population. **(A)** Lethal factor was taken in excess at a concentration of 3 µg/ml and a gradient of Protective antigen was used from concentration 0 µg/ml to 3 µg/ml. Minimum lethal concentration of Protective antigen was 1 µg/ml. **(B)** Protective antigen was taken at a concentration of 1 µg/ml and a gradient of Lethal factor was used from concentration 0 µg/ml to 3 µg/ml. Minimum lethal concentration of Lethal factor was 1.5 µg/ml.

Figure S4: Expression profiles of PA63 and PA-FL

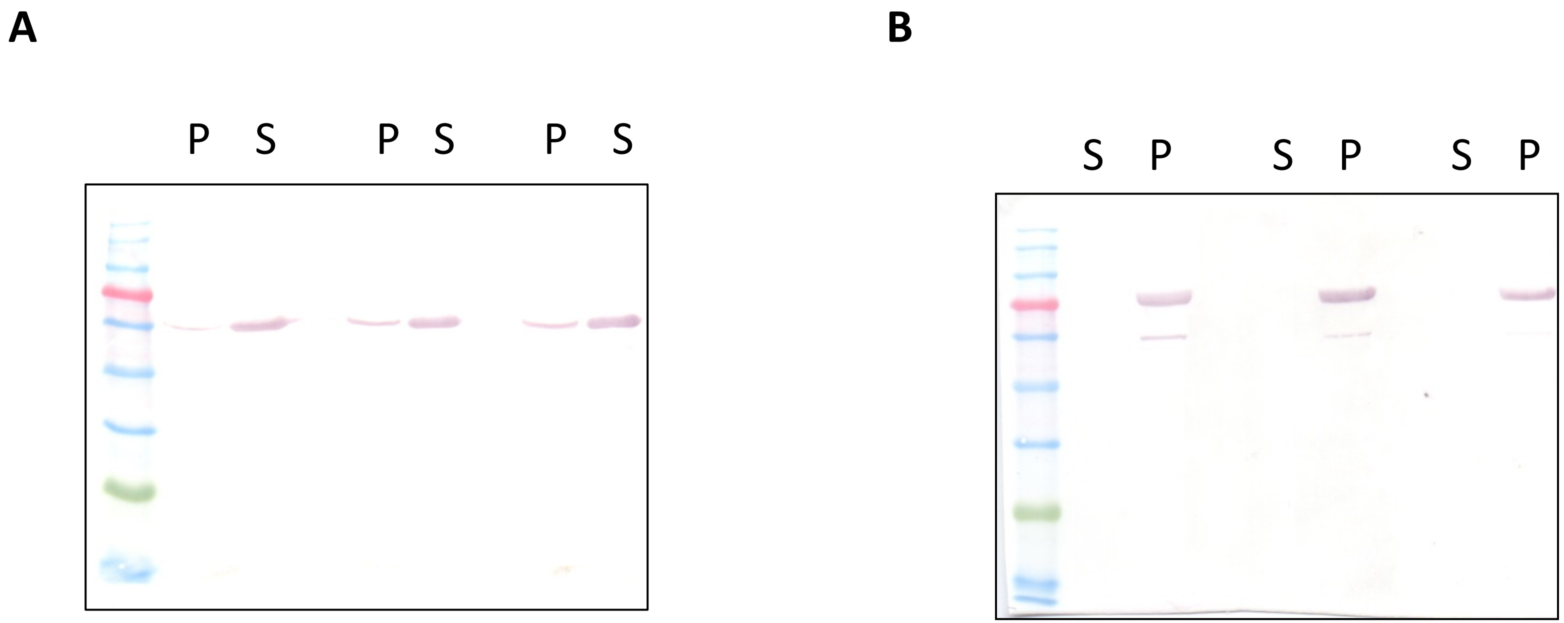

Figure S4: Immunoblotting to analyze the expression of (A) PA63 and (B) PAFL in *E. coli*. S and P represents soluble cell Fraction and inclusion bodies respectively. PA63 is getting expressed both in soluble and inclusion bodies fraction. PAFL was expressed only as inclusion bodies.
