# Supplementary material for: Recombinant full-length Bacillus Anthracis protective antigen and its 63 kDa form elicits protective response in formulation with addavax
Source: Front Immunol. 2023 Jan 12;13:1075662. doi: 10.3389/fimmu.2022.1075662 (PMC9877290; doi:10.3389/fimmu.2022.1075662)
Supplement: Supplementary file 2 [file DataSheet_2.pdf]

Supplementary Information

Table S3

Bacillus anthracis protective antigen

Protein Sequence

MEVKQENRLLNESESSSQGLLGYYFSDLNFQAPMVVTSSTTGDLSIPSSELENIPSENQY

FQSAIWSGFIKVKKSDEYTFATSADNHVTMWVDDQEVINKASNSNKIRLEKGRLYQIKIQ

YQRENPTEKGLDFKLYWTDSQNKKEVISSDNLQLPELKQKSSNSRKKRSTSAGPTVPDRD

NDGIPDSLEVEGYTVDVKNKRTFLSPWISNIHEKKGLTKYKSSPEKWSTASDPYSDFEKV

TGRIDKNVSPEARHPLVAAYPIVHVDMENIILSKNEDQSTQNTDSQTRTISKNTSTSRTH

TSEVHGNAEVHASFFDIGGSVSAGFSNSNSSTVAIDHSLSLAGERTWAETMGLNTADTAR

LNANIRYVNTGTAPIYNVLPTTSLVLGKNQTLATIKAKENQLSQILAPNNYYPSKNLAPI

ALNAQDDFSSTPITMNYNQFLELEKTKQLRLDTDQVYGNIATYNFENGRVRVDTGSNWSE

VLPQIQETTARIIFNGKDLNLVERRIAAVNPSDPLETTKPDMTLKEALKIAFGFNEPNGN

LQYQGKDITEFDFNFDQQTSQNIKNQLAELNATNIYTVLDKIKLNAKMNILIRDKRFHYD

RNNIAVGADESVVKEAHREVINSSTEGLLLNIDKDIRKILSGYIVEIEDTEGLKEVINDR

YDMLNISSLRQDGKTFIDFKKYNDKLPLYISNPNYKVNVYAVTKENTIINPSENGDTSTN

GIKKILIFSKKGYEIG

DNA Sequences

PA20

ATGGAAGTTAAACAGGAGAACCGGTTATTAAATGAATCAGAATCAAGTTCCCAGGGGTTACTAGGATACTATTTTAG
TGATTTGAATTTTCAAGCACCCATGGTGGTTACCTCTTCTACTACAGGGGATTTATCTATTCCTAGTTCTGAGTTAG
AAAATATTCCATCGGAAAACCAATATTTTCAATCTGCTATTTGGTCAGGATTTATCAAAGTTAAGAAGAGTGATGAA
TATACATTTGCTACTTCCGCTGATAATCATGTAACAATGTGGGTAGATGACCAAGAAGTGATTAATAAAGCTTCTAA
TTCTAACAAAATCAGATTAGAAAAAGGAAGATTATATCAAATAAAAATTCAATATCAACGAGAAAATCCTACTGAAA
AAGGATTGGATTTCAAGTTGTACTGGACCGATTCTCAAAATAAAAAAGAAGTGATTTCTAGTGATAACTTACAATTG
CCAGAATTAAAACAAAAATCTTCGAACTCAAGAAAAAAGCGAAGT

PA Domain I` (Domain I- PA 20)

```
ACAAGTGCTGGACCTACGGTTCCAGACCGTGACAATGATGGAATCCCTGATTCATTAGAGGTAGAAGGATATACGGT
TGATGTCAAAAATAAAAGAACTTTTCTTTCACCATGGATTTCTAATATTCATGAAAAGAAAGGATTAACCAAATATA
AATCATCTCCTGAAAAATGGAGCACGGCTTCTGATCCGTACAGTGATTTCGAAAAGGTTACAGGACGGATTGATAAG
AATGTATCACCAGAGGCAAGACACCCCCTTGTGGCAGCTTAT
```

PA 63

```
ACAAGTGCTGGACCTACGGTTCCAGACCGTGACAATGATGGAATCCCTGATTCATTAGAGGTAGAAGGATATACGGT
TGATGTCAAAAATAAAAGAACTTTTCTTTCACCATGGATTTCTAATATTCATGAAAAGAAAGGATTAACCAAATATA
AATCATCTCCTGAAAAATGGAGCACGGCTTCTGATCCGTACAGTGATTTCGAAAAGGTTACAGGACGGATTGATAAG
AATGTATCACCAGAGGCAAGACACCCCCTTGTGGCAGCTTATCCGATTGTACATGTAGATATGGAGAATATTATTCT
CTCAAAAAATGAGGATCAATCCACACAGAATACTGATAGTCAAACGAGAACAATAAGTAAAAATACTTCTACAAGTA
GGACACATACTAGTGAAGTACATGGAAATGCAGAAGTGCATGCGTCGTTCTTTGATATTGGTGGTAGTGTATCTGCA
GGATTTAGTAATTCGAATTCAAGTACGGTCGCAATTGATCATTCACTATCTCTAGCAGGGGAAAGAACTTGGGCTGA
AACAATGGGTTTAAATACCGCTGATACAGCAAGATTAAATGCCAATATTAGATATGTAAATACTGGGACGGCTCCAA
TCTACAACGTGTTACCAACGACTTCGTTAGTGTTAGGAAAAAATCAAACACTCGCGACAATTAAAGCTAAGGAAAAC
CAATTAAGTCAAATACTTGCACCTAATAATTATTATCCTTCTAAAAACTTGGCGCCAATCGCATTAAATGCACAAGA
CGATTTCAGTTCTACTCCAATTACAATGAATTACAATCAATTTCTTGAGTTAGAAAAAACGAAACAATTAAGATTAG
ATACGGATCAAGTATATGGGAATATAGCAACATACAATTTTGAAAATGGAAGAGTGAGGGTGGATACAGGCTCGAAC
TGGAGTGAAGTGTTACCGCAAATTCAAGAAACAACTGCACGTATCATTTTTAATGGAAAAGATTTAAATCTGGTAGA
AAGGCGGATAGCGGCGGTTAATCCTAGTGATCCATTAGAAACGACTAAACCGGATATGACATTAAAAGAAGCCCTTA
AAATAGCATTTGGATTTAACGAACCGAATGGAAACTTACAATATCAAGGGAAAGACATAACCGAATTTGATTTTAAT
TTCGATCAACAAACATCTCAAAATATCAAGAATCAGTTAGCGGAATTAAACGCAACTAACATATATACTGTATTAGA
TAAAATCAAATTAAATGCAAAAATGAATATTTTAATAAGAGATAAACGTTTTCATTATGATAGAAATAACATAGCAG
TTGGGGCGGATGAGTCAGTAGTTAAGGAGGCTCATAGAGAAGTAATTAATTCGTCAACAGAGGGATTATTGTTAAAT
ATTGATAAGGATATAAGAAAAATATTATCAGGTTATATTGTAGAAATTGAAGATACTGAAGGGCTTAAAGAAGTTAT
AAATGACAGATATGATATGTTGAATATTTCTAGTTTACGGCAAGATGGAAAAACATTTATAGATTTTAAAAAATATA
ATGATAAATTACCGTTATATATAAGTAATCCCAATTATAAGGTAAATGTATATGCTGTTACTAAAGAAAACACTATT
ATTAATCCTAGTGAGAATGGGGATACTAGTACCAACGGGATCAAGAAAATTTTAATCTTTTCTAAAAAAGGCTATGA
GATAGGATAA
```

Domain I

```
ATGGAAGTTAAACAGGAGAACCGGTTATTAAATGAATCAGAATCAAGTTCCCAGGGGTTACTAGGATACTATTTTAG
TGATTTGAATTTTCAAGCACCCATGGTGGTTACCTCTTCTACTACAGGGGATTTATCTATTCCTAGTTCTGAGTTAG
AAAATATTCCATCGGAAAACCAATATTTTCAATCTGCTATTTGGTCAGGATTTATCAAAGTTAAGAAGAGTGATGAA
TATACATTTGCTACTTCCGCTGATAATCATGTAACAATGTGGGTAGATGACCAAGAAGTGATTAATAAAGCTTCTAA
TTCTAACAAAATCAGATTAGAAAAAGGAAGATTATATCAAATAAAAATTCAATATCAACGAGAAAATCCTACTGAAA
AAGGATTGGATTTCAAGTTGTACTGGACCGATTCTCAAAATAAAAAAGAAGTGATTTCTAGTGATAACTTACAATTG
CCAGAATTAAAACAAAAATCTTCGAACTCAAGAAAAAAGCGAAGTACAAGTGCTGGACCTACGGTTCCAGACCGTGA
CAATGATGGAATCCCTGATTCATTAGAGGTAGAAGGATATACGGTTGATGTCAAAAATAAAAGAACTTTTCTTTCAC
CATGGATTTCTAATATTCATGAAAAGAAAGGATTAACCAAATATAAATCATCTCCTGAAAAATGGAGCACGGCTTCT
GATCCGTACAGTGATTTCGAAAAGGTTACAGGACGGATTGATAAGAATGTATCACCAGAGGCAAGACACCCCCTTGT
GGCAGCTTAT
```

Domain II

CCGATTGTACATGTAGATATGGAGAATATTATTCTCTCAAAAAATGAGGATCAATCCACACAGAATACTGATAGTCA
AACGAGAACAATAAGTAAAAATACTTCTACAAGTAGGACACATACTAGTGAAGTACATGGAAATGCAGAAGTGCATG
CGTCGTTCTTTGATATTGGTGGTAGTGTATCTGCAGGATTTAGTAATTCGAATTCAAGTACGGTCGCAATTGATCAT
TCACTATCTCTAGCAGGGGAAAGAACTTGGGCTGAAACAATGGGTTTAAATACCGCTGATACAGCAAGATTAAATGC
CAATATTAGATATGTAAATACTGGGACGGCTCCAATCTACAACGTGTTACCAACGACTTCGTTAGTGTTAGGAAAAA
ATCAAACACTCGCGACAATTAAAGCTAAGGAAAACCAATTAAGTCAAATACTTGCACCTAATAATTATTATCCTTCT
AAAAACTTGGCGCCAATCGCATTAAATGCACAAGACGATTTCAGTTCTACTCCAATTACAATGAATTACAATCAATT
TCTTGAGTTAGAAAAAACGAAACAATTAAGATTAGATACGGATCAAGTATATGGGAATATAGCAACATACAATTTTG
AAAATGGAAGAGTGAGGGTGGATACAGGCTCGAACTGGAGTGAAGTGTTACCGCAAATTCAAGAAACAACT

Domain III

GCACGTATCATTTTTAATGGAAAAGATTTAAATCTGGTAGAAAGGCGGATAGCGGCGGTTAATCCTAGTGATCCATT
AGAAACGACTAAACCGGATATGACATTAAAAGAAGCCCTTAAAATAGCATTTGGATTTAACGAACCGAATGGAAACT
TACAATATCAAGGGAAAGACATAACCGAATTTGATTTTAATTTCGATCAACAAACATCTCAAAATATCAAGAATCAG
TTAGCGGAATTAAACGCAACTAACATATATACTGTATTAGATAAAATCAAATTAAATGCAAAAATGAATATTTTAAT
AAGAGATAAACGTTTT

Domain IV

CATTATGATAGAAATAACATAGCAGTTGGGGCGGATGAGTCAGTAGTTAAGGAGGCTCATAGAGAAGTAATTAATTC
GTCAACAGAGGGATTATTGTTAAATATTGATAAGGATATAAGAAAAATATTATCAGGTTATATTGTAGAAATTGAAG
ATACTGAAGGGCTTAAAGAAGTTATAAATGACAGATATGATATGTTGAATATTTCTAGTTTACGGCAAGATGGAAAA
ACATTTATAGATTTTAAAAAATATAATGATAAATTACCGTTATATATAAGTAATCCCAATTATAAGGTAAATGTATA
TGCTGTTACTAAAGAAAACACTATTATTAATCCTAGTGAGAATGGGGATACTAGTACCAACGGGATCAAGAAAATTT
TAATCTTTTCTAAAAAAGGCTATGAGATAGGATAA

D1+D IV

ATGGAAGTTAAACAGGAGAACCGGTTATTAAATGAATCAGAATCAAGTTCCCAGGGGTTACTAGGATACTATTTTAG
TGATTTGAATTTTCAAGCACCCATGGTGGTTACCTCTTCTACTACAGGGGATTTATCTATTCCTAGTTCTGAGTTAG
AAAATATTCCATCGGAAAACCAATATTTTCAATCTGCTATTTGGTCAGGATTTATCAAAGTTAAGAAGAGTGATGAA
TATACATTTGCTACTTCCGCTGATAATCATGTAACAATGTGGGTAGATGACCAAGAAGTGATTAATAAAGCTTCTAA
TTCTAACAAAATCAGATTAGAAAAAGGAAGATTATATCAAATAAAAATTCAATATCAACGAGAAAATCCTACTGAAA
AAGGATTGGATTTCAAGTTGTACTGGACCGATTCTCAAAATAAAAAAGAAGTGATTTCTAGTGATAACTTACAATTG
CCAGAATTAAAACAAAAATCTTCGAACTCAAGAAAAAAGCGAAGTACAAGTGCTGGACCTACGGTTCCAGACCGTGA
CAATGATGGAATCCCTGATTCATTAGAGGTAGAAGGATATACGGTTGATGTCAAAAATAAAAGAACTTTTCTTTCAC
CATGGATTTCTAATATTCATGAAAAGAAAGGATTAACCAAATATAAATCATCTCCTGAAAAATGGAGCACGGCTTCT
GATCCGTACAGTGATTTCGAAAAGGTTACAGGACGGATTGATAAGAATGTATCACCAGAGGCAAGACACCCCCTTGT
GGCAGCTTATGGATCCCATTATGATAGAAATAACATAGCAGTTGGGGCGGATGAGTCAGTAGTTAAGGAGGCTCATA
GAGAAGTAATTAATTCGTCAACAGAGGGATTATTGTTAAATATTGATAAGGATATAAGAAAAATATTATCAGGTTAT
ATTGTAGAAATTGAAGATACTGAAGGGCTTAAAGAAGTTATAAATGACAGATATGATATGTTGAATATTTCTAGTTT
ACGGCAAGATGGAAAAACATTTATAGATTTTAAAAAATATAATGATAAATTACCGTTATATATAAGTAATCCCAATT

ATAAGGTAAATGTATATGCTGTTACTAAAGAAAACACTATTATTAATCCTAGTGAGAATGGGGATACTAGTACCAAC
GGGATCAAGAAAATTTTAATCTTTTCTAAAAAAGGCTATGAGATAGGATAA
